# Development of a high-resolution infrared thermographic imaging method as a diagnostic tool for acute undifferentiated limp in young children

**DOI:** 10.1007/s11517-017-1749-0

**Published:** 2017-11-28

**Authors:** R. Owen, S. Ramlakhan, R. Saatchi, D. Burke

**Affiliations:** 1 The University of Sheffield Medical School, Sheffield, UK; 2 Sheffield Children’s NHS Foundation Trust, Sheffield, UK; 3 Materials and Engineering Research Institute, Sheffield Hallam University, Sheffield, UK; 4 Department of Clinical Surgical Sciences, University of the West Indies, St. Augustine, W.I. Trinidad and Tobago

**Keywords:** Thermography, Limp diagnosis, Leg injuries, Children

## Abstract

Acute limp is a common presenting condition in the paediatric emergency department. There are a number of causes of acute limp that include traumatic injury, infection and malignancy. These causes in young children are not easily distinguished. In this pilot study, an infrared thermographic imaging technique to diagnose acute undifferentiated limp in young children was developed. Following required ethics approval, 30 children (mean age = 5.2 years, standard deviation = 3.3 years) were recruited. The exposed lower limbs of participants were imaged using a high-resolution thermal camera. Using predefined regions of interest (ROI), any skin surface temperature difference between the healthy and affected legs was statistically analysed, with the aim of identifying limp. In all examined ROIs, the median skin surface temperature for the affected limb was higher than that of the healthy limb. The small sample size recruited for each group, however, meant that the statistical tests of significant difference need to be interpreted in this context. Thermal imaging showed potential in helping with the diagnosis of acute limp in children. Repeating a similar study with a larger sample size will be beneficial to establish reproducibility of the results.

**Graphical abstract:**
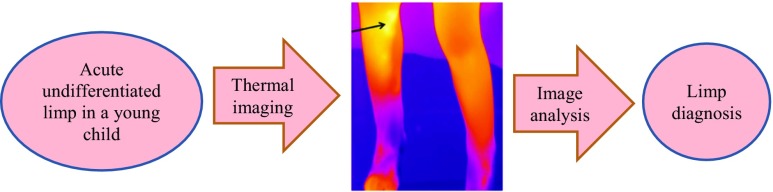
A young child with an acute undifferentiated limp undergoes thermal imaging and the follow on image analysis assists the limp diagnosis.

A young child with an acute undifferentiated limp undergoes thermal imaging and the follow on image analysis assists the limp diagnosis.

## Introduction

Acute limp is a common presenting complaint in the paediatric emergency department (ED). A UK study reported the incidence of a non-traumatic limp as 1.8 in every 1000 children aged under 14 years, but the true incidence representing all limp is much higher [1]. An ‘acute’ limp is defined in a patient presenting with symptoms of recent onset, often in the last 48 hours. An ‘undifferentiated’ limp is not yet specified by clinical diagnosis and requires both physical examination and further investigation to determine the cause of the complaint.

Limp in children is often caused by a traumatic injury, but may also result from minor inflammatory conditions or more serious infective causes. In many children, the definitive diagnosis may not be easy to establish, especially as young children are often unable to indicate the region of pain. Several differential diagnoses for acute limp have been known to have associated skin surface temperature changes; therefore, high-resolution infrared thermography (IRT) presents an additional examination tool for this medical condition.

In an environment such as the ED, where prompt diagnosis and efficient management of patients are essential, IRT has the potential to improve the assessment of acute limp. Particularly in young children, in which it may be difficult to identify the specific region of pathology in an affected limb, thermography may be able to highlight a suspected region of interest to allow for more focussed investigation.

There is an ongoing debate as to whether or not the doses of ionizing radiation that are used in medical X-ray imaging do increase the risk of cancer [2]. Most of the hypothetical increased cancer risk from medical radiation exposure comes from CT and nuclear medicine, not from X-ray radiographs. Furthermore, among radiographs, those of the extremities have the lesser radiation effective dose. The ratio of positive to negative radiographs is facility provider and pathology (fracture, for example) dependent. Given these issues, a reduction to exposure to X-ray that could result from the use of IRT is a positive development.

Infrared radiation is naturally emitted from any source of heat, including the human body, allowing images to be produced as ‘temperature maps’. These images can be conveniently produced by a thermal camera, which may be used to detect areas of increased or decreased temperature on particular areas of skin surface. IRT has been used in industry as an effective investigative diagnostic tool for decades, but its applications in the medical field are emerging, with improvements in the quality and availability of highly sensitive thermal cameras and advanced thermal image processing techniques. This study aimed to explore a unique aspect of medical thermography, with a focus on a paediatric emergency environment.

Many pathological conditions cause inflammatory reactions that result in localised vascular changes around the affected areas of the body. The blood and vasculature act as a heat-transferring mechanism for the body, conducting heat produced as a product of metabolism. In this way, some medical conditions can cause localised changes in the skin surface temperature, which can indicate the presence of an abnormality, or even its severity. By accurately measuring the temperature of the relevant areas of the body, IRT may be able to quantify the progression or severity of particular abnormalities.

IRT has been used in adult medicine for a number of conditions, using skin temperature changes for diagnosis and monitoring. From its first recorded medical usage in screening rheumatoid joints in adults, IRT has been shown effective in detecting joint inflammation in a number of subsequent studies [3–6]. Similarly, IRT has been shown to detect changes in blood supply associated with peripheral vascular disease, as well as detecting peripheral neuropathy in diabetic patients [7–9].

In children, previous studies have shown IRT to be an effective tool in respiratory monitoring, using nasal temperature differentials determined through image tracking systems [10–13]. Additionally, IRT has been successfully applied to the diagnosis of varying degrees of paediatric skin burn, with a reported sensitivity and specificity often greater than that of clinical assessment [14, 15]. Recent pilot studies have shown that IRT can have other applications in paediatric emergency medicine, in screening for specific conditions such as fracture and joint inflammation, as well as monitoring wounds for signs of healing or infection [15–18, 29]. In a prospective study of 51 children presenting with unilateral limb injury, the radiographs and thermal images were analysed; 11 showed fracture on radiograph, of which, 7 (64%) were matched by thermal imaging [19]. Thermal imaging matched the site of pain in 36 out of 49 (73%) examined limbs. A number of studies have suggested that IRT to be more effective in a younger population, with reduced variability in skin temperature measurements and more responsive vascular systems to cold challenge testing [20, 21].

Drawing from information presented in the literature, this study aimed to develop and evaluate a method of infrared thermographic imaging to assist diagnosis of acute undifferentiated limp in young children. As part of realising this aim, the study required the following:

i. Design of a suitable protocol to record thermal imaging data and recruit an appropriate sample of patients, following the UK’s National Health Service (NHS) ethical approval
ii. Temperature measurement of targeted regions of the lower limbs, in children presenting with an acute limp to the emergency department and devise a method of processing for the recorded images
iii. Analysis and interpretation of the results in conjunction with clinical notes, comparing the affected side with the healthy side (acting as a control)

In the following sections, the developed methodology, results and their discussions and conclusions are provided.

## Methodology

### Ethical approval

Ethical approval for the study was obtained from the NHS Leeds West Research Ethics Committee (REC) (REC reference number 15/YH/0562). All parents of the children included in this study signed the informed consent form at the time of recruitment.

### Data collection

Thirty patients were sequentially recruited from the Sheffield Children’s NHS Foundation Trust (Sheffield Children’s Hospital, Sheffield, U.K.) Emergency Department (ED) over a period of 2 months (02/2016–03/2016). None of the 30 recruited patients were excluded. All patients were compliant. Ten patients did not receive medication, 13 received paracetamol, 3 received ibuprofen and 4 received both paracetamol and ibuprofen. Due to the variations in the medication and the small sample size, there was no correlation between receiving anti-inflammatory drugs and the results of thermal imaging.

The recruited patients had only one leg affected by limp, allowing the unaffected healthy leg to act as a temperature reference. Patients were included in the study if they were aged less than 15 years and presented with an acute limp. The inclusion criteria were deliberately broad to include a variety of presenting complaints, so the application of IRT could be assessed across a number of different related conditions. To improve consistency of analysis, patients were excluded if more than 48 h had passed since the onset of the symptoms or injury. The patients were also excluded if they (or their parents) refused to sign the consent form or the child was not comfortable in taking part in the study.

All infrared thermal image recordings were carried out in the same treatment room in the Sheffield Children’s NHS Foundation Trust ED, which had a consistent temperature and humidity. The room had a window, but its blinds were shut to obstruct direct sunlight and there was no source of draught, which could influence the recordings. The room temperature and humidity were monitored for each recording. The company who provided the thermal camera also organised comprehensive training to the team on how best to deploy the camera. All recordings were made by the same researcher to ensure consistency. The instructions given to the participating children, the recording procedure and environment were maintained as consistent as possible.

The child was required to remove all clothing below the waist, excluding underwear, and was seated on a stool in the middle of the recording room. A towel was placed on the stool, to thermally insulate the child from the stool as much as possible. In this position, the region to be imaged was allowed to acclimatise for 10 min, in keeping with the practice of previous studies [22]. The child was instructed not to touch the areas that were intended for imaging during the acclimatisation and recording.

Each patient’s relevant details, including date of birth, height, weight, gender and body temperature, were recorded along with the mechanism and location of injury. Also, the patient’s hospital number was noted to allow follow-up information and diagnosis to be obtained; however, this was encoded on a separate identifier sheet to ensure anonymity.

During consultation with a qualified ED doctor, patients presenting with an acute limp that were eligible for the study were brought to the attention of the researcher. In order to diagnose such patients, investigations such as X-ray imaging or ultrasound scan were conducted as necessary to supplement the clinical examination and aid the clinician’s judgement. So as to avoid any delay in the patient’s treatment, data collection for the study took place when patients waited for investigation results. In order to ensure an unbiased investigation, the clinicians involved in recruiting and diagnosis were blinded to the results obtained from the thermal imaging, and the researcher that performed the thermal imaging analysis was blinded to the medical diagnostic results. A third researcher independent of the clinician and the person who carried out the thermal image processing statistically compared the medical diagnosis and thermal imaging findings.

The infrared camera was mounted on an advanced tripod that allowed the camera to be accurately positioned to the required heights and orientations. The distance between the camera lens and the patient was kept to 1 m in all recordings. There is not a standard predefined distance for thermal imaging due to variations in the camera specifications and applications’ requirements. When the camera is brought closer to the subject, the region of interest is represented by a larger number of pixels thus enhancing the accuracy of the analysis. However, issues including the camera’s optimum focus, zoom and practicalities such as ensuring an adequate distance from the child to avoid disturbing him/her limit the closeness of the camera to the subject. In our previous studies involving thermal imaging from children, a distance of 1 m proved effective [10]. Instead of recording a single image, a 20-s video recording was obtained, repeated with the patient in four different positions, i.e. anterior sitting, anterior standing, left lateral standing and right lateral standing. All recordings were repeated in both sitting and standing positions because it was not known which mode gave improved results.

**Fig. 1:**
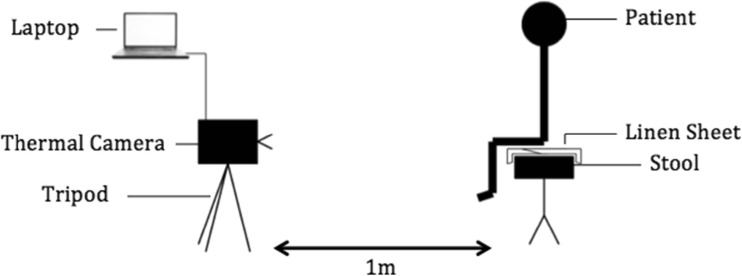
A schematic diagram of the experimental set up

**Fig. 2:**
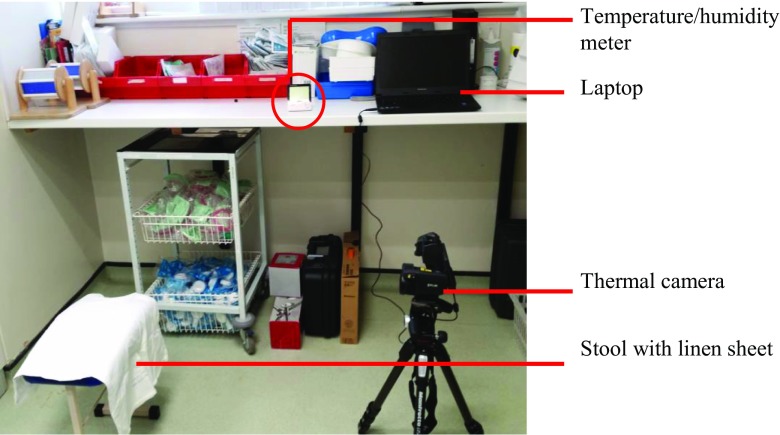
Photograph of the experimental setup

The video was recorded at a rate of 30 Hz (30 frames per second), producing 600 images per video recording over 20 s. The tripod remained in the same location each time, but the tripod’s head was suitably adjusted in the vertical plane to capture the desired region of interest. This procedure ensured a standardised and easily reproducible method of infrared thermal video recording. A diagram of the setup for the data recording is provided in Fig. 1 whilst Fig. 2 shows a photograph of the actual setup.

### Materials

The choice of camera would have affected the accuracy of the results obtained in the study. In this study, an FLIR© T630sc thermal camera (FLIR Systems UK, West Malling) and its associated FLIR Research Max 4© software were used to collect the thermographic infrared videos. Table 1 provides the features of this FLIR© T630sc thermal camera. The camera had a sufficiently low noise equivalent temperature difference, a measure of its thermal sensitivity, (less than 0.04 K) and image resolution (640 × 480 pixels). Its image capture rate was up to 30 frames per second, which was sufficient for this study. The cameras used in other related studies were comparable. For example, to study the thermal symmetry of the upper and lower extremities in healthy subjects, an FLIR A40 thermal camera was used [23]. The image resolution and noise equivalent temperature difference for this camera are 0.08 K and 320 × 240, respectively. An FLIR SC300 thermal camera was used to detect inflammation in thyroid eye disease [24]. The image resolution and noise equivalent temperature difference for this camera are 320 × 240 and less than 0.05 K, respectively. An FLIR SC500 camera was used to evaluate the symmetry of muscle activity in different exercises [25]. The image resolution and noise equivalent temperature difference for this camera are 320 × 240 and less than 0.1 K, respectively.

**Table 1:** Features of the FLIR© T630sc thermal camera

Feature	Specification
Detector type	Uncooled microbolometer
Spectral range	7.5–13.0 μm
Image resolution	640 × 480 pixels
Detector pitch 25 μm	17 μm
Noise equivalent temperature difference (a measure of camera’s thermal sensitivity)	< 40 mK
Maximum capture rate	30 Hz
Standard temperature range	−40 to 650 °C
Accuracy	+/−2 °C +/−2%

The video and image processing was carried out using Matlab© package.

Patient information and diagnosis results were tabulated using Microsoft Excel© spreadsheet (Microsoft Corporation). These data were then processed and analysed, along with the results from the thermal image processing, using IBM© SPSS© software (IBM© Corporation).

### Image processing

**Fig. 3:**
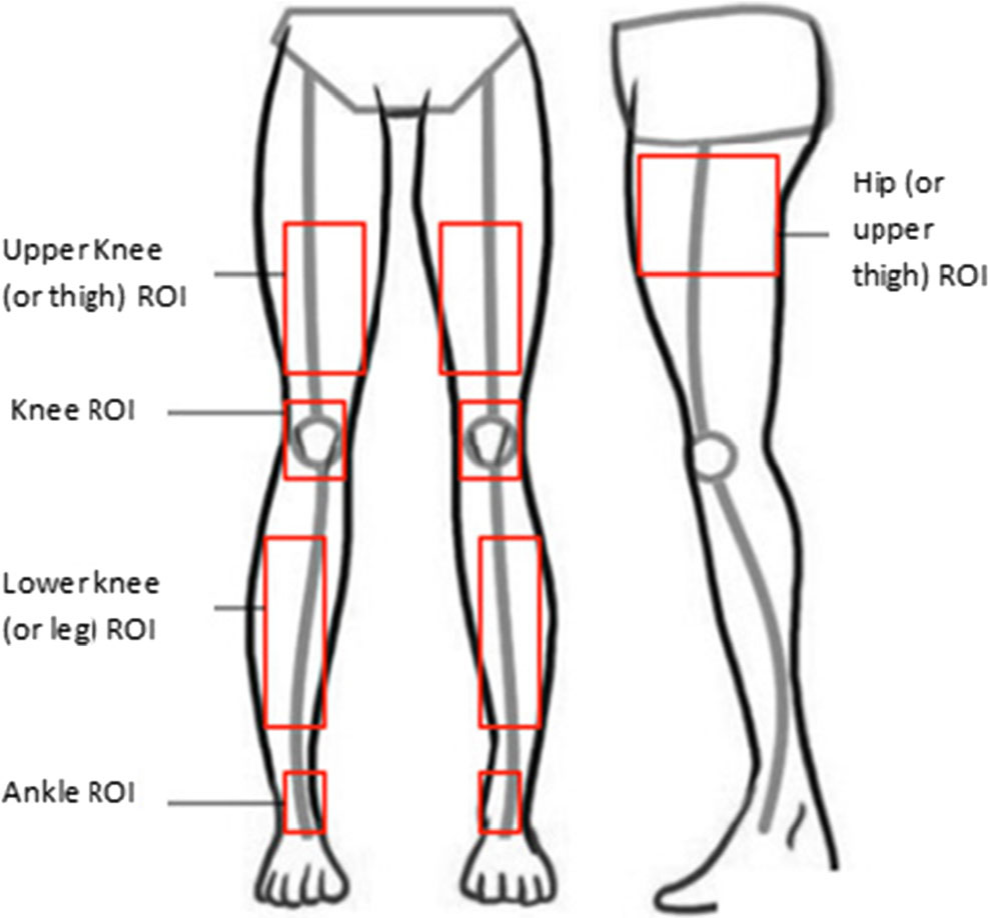
Illustration of the locations of ROIs for processing of the thermal images

Five regions of interest (ROI) on the legs were considered as shown in Fig. 3. These corresponded to (i) upper knee (front side), (ii) the knee (front side), (iii) lower knee (front side), (iv) ankle (front side) and (v) hip. The hip could not be reliably imaged in the frontal view because of the temperature effects of underwear (for ethical reasons the participants kept their underwear on for the measurements). The lateral view of this region was more freely accessible, so for the hip, the lateral ROI was chosen. For the other ROIs, this was not an issue and frontal views were used.

Each region was individually analysed by comparing the skin surface temperature of its associated ROI on both the affected and unaffected (healthy) legs. For each region, the related thermal video footage was visually scanned and 20 best frames were selected for processing. This selection was based on the image angle of the patient (in some frames the patient had moved, making the region of analysis not adequately visible in the image and so these images were not used). The analysis results were averaged across these 20 images.

The selection of ROIs was consistent for all the patients analysed and reproduced as accurately as possible. The sizes of the five ROIs varied for each patient and for each affected region. The patients with larger legs had larger ROIs, whilst the ROI for the lower knee was larger than that of the knee region. For an individual patient, the size of an ROI for a specific region on the affected leg was identical to the corresponding ROI on the healthy leg. The related locations also closely matched.

Each ROI was selected manually to include as much as possible of the affected region. This was a compromise between selecting a larger region that was more representative and smaller region that allowed a more focused analysis. Once an ROI on affected leg was selected, an exact size and location was chosen on the healthy leg to act as its reference. The operation to determine the coordinates and size of the ROI relied on the Matlab© graphic image processing user interface. This involved displaying the required image on a large computer screen and manually placing a rectangle on the required location. Then using a mouse, the pixel coordinates of the top left corner and bottom right corner of the square were read from the image. By entering these coordinates to a Maltab© function, the ROI with the required location and size was cropped from the rest of the image. The correct selection of ROI is an important issue in thermography. The manner we selected and the ROI is similar to a number of studies that used thermal imaging in medical diagnosis. For example, in a study that investigated the effectiveness of thermography in detecting lower extremity deep venous thrombosis, areas of interest on the left and right leg were identified and the mean temperature of the pixels within the regions were obtained [26]. In another study that used thermal imaging to examine body surface temperature on tetraplegic and paraplegic patients, square regions of interest on the body and the averages of the pixel values in those regions were analysed [27].

The following procedure was followed to determine and analyse the temperature of an ROI for the affected leg, along with its reference ROI on the healthy leg.

i. The pixel values (representing the location’s temperature) within the ROI were averaged to obtain a single mean value, representing the temperature of the ROI in a frame.
ii. The operation (i) was repeated for the 20 selected frames for each patient.
iii. The resulting 20 temperature values were further averaged to obtain a pair of mean values representing the ROI’s temperatures for the affected and healthy legs for each patient.

The above operations resulted in five pairs of averaged temperature values for the five ROIs of the affected and healthy legs.

No formal assessment of the thermal imaging tolerability was performed. However, prior to the data recordings, a brief explanation of technology was provided and the children generally found the manner with which heat from the body can be visualised interesting (further encouraging to engage) and all participants were fully compliant in the data collection.

### Statistical analysis

Prior to the statistical analysis, the patient data were organised into three groups based on the clinical diagnosis that had been carried by the patient’s medical consultant. These were soft tissue injury, irritable hip and fracture.

For clinically relevant results, the participants were divided by presenting complaint regions, so that the analysis of the skin surface temperature could be focused on the specific region affected by pathology. The presenting complaint regions were hip and thigh, knee, lower leg and ankle. In this way, only the relevant ROI measurements were included in the analysis, for each presenting complaint region. For example, in patients with pain in the hip or thigh, the hip and upper knee ROIs were included in the analysis.

As the sample size for each of these groups was small, the distribution of the mean temperatures of the ROIs across subjects was often skewed. Therefore, it was more representative to use median values when comparing the results across subjects. In addition to descriptive statistics, the percentage temperature difference was used to compare the affected side with the unaffected side.

## Results

The demographics for the 30 patients recruited to this pilot study are included in Table 2. As patients were recruited from the ED, the injuries had varied mechanisms, as indicated in Table 3.

**Table 2:** Patients’ data demographics

Parameter	Parameter values
Mean age and standard deviation (SD) (years)	5.2 (3.0)
Sex (%)	
Female	10 (33.3%)
Male	20 (66.7%)
Mean weight and its SD (kg)	22.9 (10.1)
Mean height and its SD (cm)	115.7 (19.4)
Mean bone mass index and its SD (kg/m^2^)	17.9 (1.9)
Mean body temperature and its SD (°C)	36.2 (0.4)

**Table 3:** Distribution of presenting complaints

Feature of presenting complaint	Study participants (*n* = 30)
Trauma (%)	
Yes	16 (53.3)
No	14 (46.7)
Weight-bearing (%)	
Yes	17 (56.7)
No	13 (43.3)
Side affected (%)	
Right	11 (36.7)
Left	18 (60.0)
Both	1 (3.3)
Region affected (%)	
Hip	5 (16.7)
Thigh	8 (26.7)
Knee	6 (20.0)
Lower leg	8 (26.7)
Ankle	3 (10.0)
Diagnosis (%)	
Soft tissue injury	13 (43.3)
Irritable hip	9 (30.0)
Toddler’s fracture	3 (10.0)
Other	4 (13.3)
Unknown diagnosis	1 (3.3)

The diagnoses described in Table 3 indicate that in the majority of recruited patients, the cause of their acute limp was a soft tissue injury. This reflects the reality of the paediatric ED, but as this pilot study aimed to apply thermal imaging to the identification of serious pathology, this majority cohort of minor injuries proved to be one of the limitations of this project. Included in the “Other” category of clinical diagnoses were one Cozen fracture, one buckle fracture, one distal tibial bony irregularity and one case of myositis. The patient affected by myositis had both legs involved and, as the statistical analysis focused on comparing the comparable regions on the affected and healthy legs, this patient was excluded from the results. Finally, one patient was lost to follow-up and a definitive diagnosis could not be made, so this participant was also excluded from the results.

When exploring a new clinical screening tool, it is important to assess the process in the context of a real-life scenario. In this case, the thermal imaging was to be assessed for its application in the management of acute undifferentiated limp in the paediatric ED. For the purposes of the statistical analysis, patients were divided by both their diagnoses and the region reportedly affected. In this way, the results from our analysis would confer a more realistic depiction of the efficacy of thermal imaging in this context.

Initial analysis revealed that the skin surface temperature of the leg tended to be highest in the hip and thigh regions, gradually decreasing towards the ankle. Due to this natural variation, different areas of the leg could not be reliably compared with each other for differences in skin surface temperature. Therefore, results were focussed on the specific region affected by pathology, with only the relevant ROI measurements used to assess whether there was a difference in skin surface temperature between the affected and healthy legs.

The results from this pilot study are summarised in Table 4. As the subgroups used in the analysis were very small, the data were heavily skewed and the median temperature values, with associated interquartile range, were more appropriate for comparison. Percentage difference was calculated using Eq. 1.

1 $$ \mathrm{percentage}\kern0.17em \mathrm{difference}=\frac{\mathrm{affected}\;\mathrm{ROI}-\mathrm{unaffected}\;\mathrm{ROI}\;\left(\mathrm{healthy}\;\mathrm{leg}\right)}{\mathrm{affected}\;\mathrm{ROI}}\times 100 $$

**Table 4:** Median temperatures of the ROIs on the affected and healthy legs. Patients are organised by diagnostic groups

Region affected by pathology	ROI measurement	Diagnostic group	Median temperature reading (°C) (interquartile range)		Percentage difference (affected to unaffected)
			Affected leg	Unaffected (healthy) leg	
Hip and thigh	Upper knee (standing position)	Soft tissue injury (*n* = 3)	32.01 (1.92)	31.18 (2.64)	2.59
		Irritable hip (*n* = 9)	32.77 (1.74)	32.54 (1.85)	0.70
	Hip (standing position)	Soft tissue injury (*n* = 4)	31.93 (1.64)	31.50 (1.12)	1.35
		Irritable hip (*n* = 8)	32.16 (2.19)	31.82 (1.60)	1.06
	Upper knee (sitting position)	Soft tissue injury (*n* = 1)	31.91 (n/a)	30.65 (n/a)	3.95
		Irritable hip (*n* = 6)	32.99 (2.49)	32.73 (1.79)	0.79
Knee	Knee (standing position)	Soft tissue injury (*n* = 4)	31.87 (1.05)	31.47 (1.80)	1.26
	Knee (sitting position)	Soft tissue injury (*n* = 6)	32.76 (1.70)	32.43 (0.99)	1.01
Lower leg	Lower knee (standing position)	Soft tissue injury (*n* = 1)	33.34 (n/a)	32.85 (n/a)	1.47
		Fracture (*n* = 3)	30.58 (2.55)	29.39 (2.08)	3.89
	Lower knee (sitting position)	Soft tissue injury (*n* = 2)	33.97 (0.38)	33.45 (0.65)	1.53
		Fracture (*n* = 4)	32.45 (3.16)	30.72 (1.92)	5.33

The results showed that across each ROI measurement included in the analysis, the affected side had a higher median temperature than the comparable location on the healthy leg. Although these results are helpful in the detection of pathology, the small sample size and high variability in the data set precluded any firm conclusions at this stage.

**Fig. 4:**
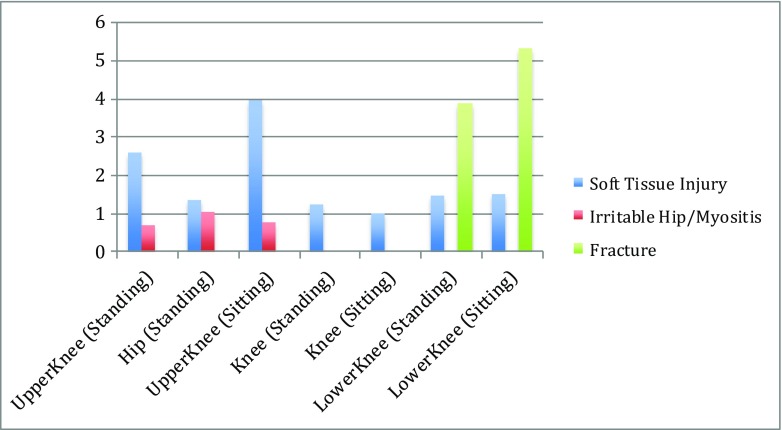
Bar chart displaying the percentage difference in median temperature, from affected leg to the healthy leg (vertical axis), for the selected ROIs and diagnoses

Figure 4 depicts the information reported in Table 4, using the percentage difference in median temperature from affected to unaffected side to display results as a bar chart.

The percentage difference for each ROI measurement, displayed in Fig. 4, highlights the variability of this data set. The most pronounced difference in temperature from the affected to healthy side was observed in the fracture group, in the sitting position. Additionally, the fracture group indicated a pronounced temperature difference in the standing position, particularly when the results are compared to the soft tissue injury group in the same ROI location. Across all ROI measurements, the irritable hip group produced the smallest percentage difference from affected to unaffected side.

### Case examples

**Fig. 5:**
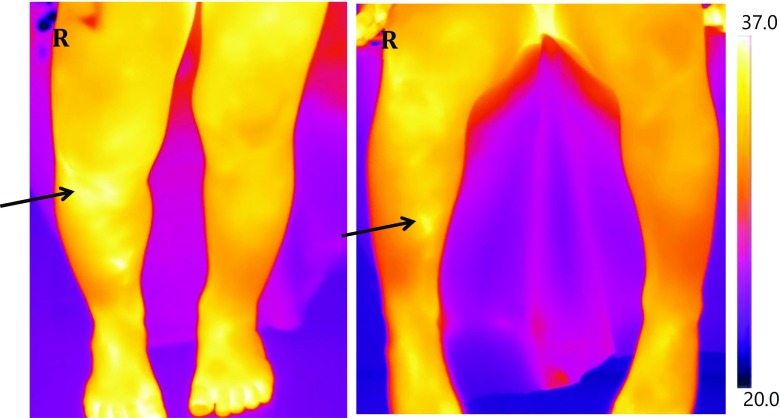
Thermal images of a patient with a toddler’s fracture of the right tibia. An area of increased temperature can be observed on the right leg (arrowed). The image on the left is taken with the patient in standing position and the right in sitting position

Figure 5 depicts an example of the thermal images produced in this study.

Figure 5 shows the thermal images taken of a boy aged 3 years that presented to the ED with acute pain in his right leg. Although no pathology was seen by the paediatric radiologist on the initial X-ray imaging, he was treated with a full-leg cast. Follow-up X-ray imaging, 2 weeks later, revealed evidence of bone healing that retrospectively confirmed a diagnosis of toddler’s fracture of the right tibia. Thermal recordings were made at the initial presentation. In the standing position (left image in Fig. 5), the lower knee ROI had a temperature value of 31.51 °C on the affected side, compared to 31.25 °C on the healthy leg. Similarly, in the sitting position (right image in Fig. 5), the lower knee ROI on the affected side had a temperature of 33.26 °C, compared to 32.37 °C in the healthy leg. This highlights a potential benefit of thermal imaging, in which, a ‘hotspot’ was observed in the area affected by pathology before other methods of investigation could detect disease.

**Fig. 6:**
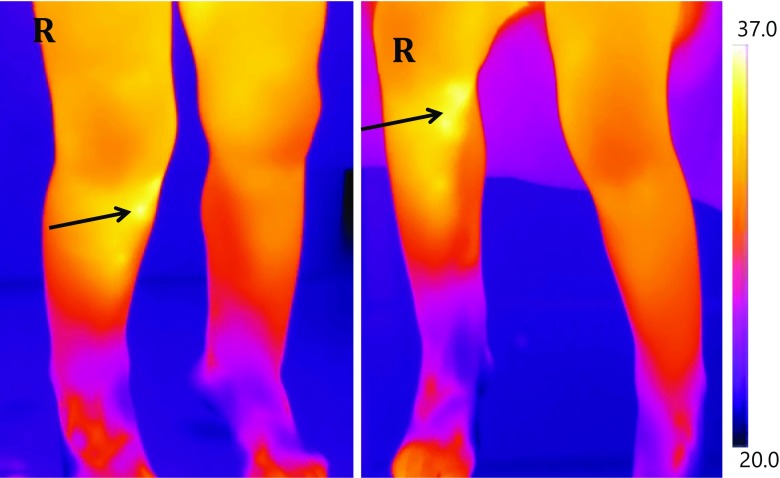
Thermal image of a patient with a Cozen fracture of the right tibia. An area of increased temperature can be observed on the right leg (arrowed). The image on the left is taken with the patient in standing position and the right in sitting position

Figure 6 depicts another example of the thermal images produced throughout this research project.

The patient displayed in Fig. 6 was a boy aged 22 months that also presented to the ED with an acute limp. This patient was diagnosed as having a Cozen fracture of the right tibia, visible on plain X-ray imaging. In the standing position (left image in Fig. 6), the lower knee ROI on the affected leg was 30.58 °C, compared with 29.17 °C on the healthy leg. Likewise, in the sitting position (right image in Fig. 6), the lower knee ROI in the affected leg was 31.64 °C, compared to 31.28 °C in the healthy leg.

## Discussion

Across each presenting complaint, the relevant ROI measurements indicated that the affected leg had a higher median temperature than the healthy leg. This was the case regardless of pathology, with every diagnostic group displaying consistent results. This would lead to the conclusion that the affected leg has an area of inflammation or injury that causes the localised skin temperature to measurably increase as compared with the same region of the healthy leg.

This outcome reiterates conclusions drawn from the previous studies that indicated inflamed or injured areas of the body are associated with a higher skin surface temperature. The study by Lasanen et al. suggested that inflamed joints had higher regional temperatures, whilst in the study involving paediatric fracture, Sanchis-Sánchez et al. showed such pathology could be detected by identifying areas of increased skin temperature [16, 17]. Conversely, a study into sports injuries and muscle strains found particular injuries to be associated with a localised decrease in temperature, so further research into this application of IRT may be necessary [28].

The bar chart in Fig. 4 highlights the differences in results across the different diagnostic groups, as well as the different imaging positions. Patients diagnosed with fracture displayed higher median temperature differences than patients diagnosed with either soft tissue injury or irritable hip. With results correlating with the severity of injury, this pilot study illustrates that it may be useful to further explore the applications of IRT as an assistive screening tool in paediatric lower limb fracture.

The inconclusive results for patients with a diagnosis of irritable hip suggest that IRT may not have the same potential in the management of such patients. The lack of significant skin temperature change in these patients may be due to the depth of the hip joint beneath the skin: inflammatory processes in the hip are less likely to confer as much of a skin temperature change than in other regions of the body. Alternatively, there may be limitations in imaging the hip with IRT due to the effect of underwear, which may increase the local temperature and confound results. Furthermore, irritable hip is currently well-detected by ultrasound technology, so there is less need for additional tools in the management of these patients.

More pronounced temperature differences, from the affected to unaffected (healthy leg) side, were observed in patients in the sitting position. There was a theoretical concern by the study team that in the sitting position, the proximity of the stool could interfere with results by causing areas of the legs to increase in temperature. The reason as to why the sitting position produced more definitive results is not entirely clear; however, changes in blood flow and pressure variations could be contributing factors. A more likely and simpler explanation may be that patients in the sitting position were able to stay still for longer, allowing better quality thermal images to be taken.

The fact that reliable, or even more definitive, results may be obtained in the sitting position removes the need for other imaging positions. This is an important outcome, particularly in reference to the diagnosis of children with an acute limp, in which they may struggle to weight-bear. Imaging such children in a sitting position reduces pain and improves compliance, which may also act to improve the quality of thermal images. A limitation of IRT deployed in his study was to encourage the young paediatric patients to wait for 10 min to allow for acclimatisation, without touching the areas to be recorded. Compliance with this was easier for recordings in the sitting position. There was a general compliance but in our study, we did not record the number of incidents where a young child may have inadvertently touched the regions of interest prior to the recordings. Accidental touching, although rare, did not result in the exclusion of the child from the study. Not excluding the children who may have accidently touched the regions of interest makes the method more practical as in clinical environment, it is not realistic to instruct a young child or an infant to completely avoid touching his or her body during the acclimatisation and thereafter during the recording. An area of further investigation could be to determine the minimum acclimatisation time needed and to establish the extent of the effect an accidental touching of the region of interest on the accuracy of the diagnosis. Finally, removing the need for multiple imaging positions speeds up the process of imaging, this is particularly important in an overcrowded ED, where an efficient management process is vital. This could be an important observation to consider when designing future studies.

This study is testing if (and by how much) a symptomatic segment of an affected limb is temperature-wise different to the correspondent segment on the contralateral healthy limb. However, it would be advantageous if the method pinpoints the affected region directly without it being given a predefined region of interest. Analysing which region on the limb is warmer or colder has a problem as it assumes the temperature of the whole limb is consistent and the medical condition or injury has altered the temperature of a particular region, which can thus be identified. This is an area of further research and we will explore it in the continuation of our study. However, the method as proposed in the paper could have diagnostic potential in its own right. This is because, for a limping child who has difficulty pointing to the site of the concern, the ROIs referred to in this study could be examined individually to locate the problematic site.

A study showed that IRT could identify the region affected by injury by measuring the area of highest temperature in the affected limb [19]. However, the study involved patients that had undergone trauma, where temperature difference may be more distinct. In order to include children with a non-traumatic cause of limp (including transient synovitis), our study compared affected limb with the healthy limb to identify the ROI with the greatest temperature differential. In our study, the technique of using IRT to identifying the site of injury was not explored as the patients in our study had marked heterogeneity of presenting complaints; however, this is an area that we will explore in future.

Although results from this pilot study are promising, the small sample size prevented a statistical test of significance to be performed. This limitation prevented definitive conclusions from being made, but this project has highlighted the possibility of further research into this application of IRT. In particular, IRT may have a role in the screening of patients with suspected toddler’s fracture, which are not always identified by X-ray imaging and often pose a diagnostic challenge.

The study focused on the sensitivity of thermal imaging to differentiate an ROI on the affected leg against the comparable region on the unaffected leg in patients (young children) with acute undifferentiated limp. A much larger study will be needed to determine the specificity of the method in differentiating between the various causes of limp.

## Conclusion

The aim of this pilot study was to assess infrared thermography (IRT) for its application as an additional tool in the management of children presenting to the ED with an acute non-specific limp. Similar to previously mentioned studies, results indicated that areas affected by pathology had an increased skin surface temperature that was quantifiable by IRT, with serious pathology being associated with a greater temperature change. However, as the sample size from this study was small, conclusions cannot be based on statistical significance testing and the outcome from this project supports the need for a larger, more focussed study into this application of IRT.

In an emergency environment, a rapid assessment of patients is essential and additional diagnostic aids that may improve the management of acute pathology are welcomed. Specifically relevant to paediatric medicine, this study found IRT to be an easy-to-use technology that was well received by a younger population; however, there remain shortcomings with regard to the need for acclimatisation and expectation that the child should not touch the regions of the interest during the acclimatisation and data recordings. Non-contact technologies such as IRT are better tolerated in very young children, where there is no risk of causing further discomfort.

There is an extent of radiation dosage from peripheral X-ray imaging; IRT by comparison does not emit any form of harmful radiation. There could be a benefit for appropriately incorporating thermography into clinical management pathways.

Finally, the protocol developed throughout this project is easily reproducible, lending itself to a busy ED environment. This pilot study highlighted the methods of imaging with IRT, which should allow the development of more sophisticated protocols for future studies. When imaging the lower limbs of children, a sitting position is preferable, with a greater temperature change in patients with a fracture. This information can be considered in future studies.

This novel application of IRT has much potential for the future management of children in the ED and there is considerable opportunity for further research into this field.
